# Dapagliflozin Ameliorates the Formation and Progression of Experimental Abdominal Aortic Aneurysms by Reducing Aortic Inflammation in Mice

**DOI:** 10.1155/2022/8502059

**Published:** 2022-01-28

**Authors:** Haole Liu, Panpan Wei, Weilai Fu, Congcong Xia, Yankui Li, Kangli Tian, Yafeng Li, Daxin Cheng, Jiaying Sun, Yangwei Xu, Ming Lu, Boyu Xu, Yali Zhang, Rong Wang, Weirong Wang, Baohui Xu, Enqi Liu, Sihai Zhao

**Affiliations:** ^1^Institute of Cardiovascular Science, Translational Medicine Institute, Xi'an Jiaotong University Health Science Center, Xi'an, Shaanxi 710061, China; ^2^Laboratory Animal Center, Xi'an Jiaotong University, Xi'an, Shaanxi 710061, China; ^3^Department of Vascular Surgery, The Second Hospital of Tianjin Medical University, Tianjin 300211, China; ^4^Division of Vascular Surgery, Department of Surgery, Stanford University School of Medicine, Stanford, CA 94305, USA

## Abstract

**Background:**

Dapagliflozin, a sodium glucose transporter protein-2 (SGLT-2) inhibitor, reduces the risk for cardiovascular diseases. However, the influence of dapagliflozin on nondissecting abdominal aortic aneurysms (AAAs) remains unclear.

**Methods:**

AAAs were created in male C57BL/6 mice via intra-aortic porcine pancreatic elastase (PPE) infusion. Mice were daily treated with dapagliflozin (1 or 5 mg/kg body weight) or an equal volume of vehicle through oral gavage beginning one day prior to PPE infusion for 14 days. To investigate its translational value, dapagliflozin or vehicle was also administered to mice with existing AAAs in another cohort. Aortic diameters were measured prior to (day 0 for baseline) and 14 days after PPE infusion. After sacrifice, mice aortae were collected, and following histological analyses were performed.

**Results:**

Dapagliflozin treatment significantly reduced aneurysmal aortic expansion following PPE infusion as compared to vehicle treatment especially at 5 mg/kg body weight (approximately 21% and 33% decreases in 1 and 5 mg/kg treatment groups, respectively). The dose-dependent attenuation of AAAs by dapagliflozin was also confirmed on histological analyses. Dapagliflozin remarkably reduced aortic accumulation of macrophages, CD4^+^ T cells, and B cells particularly following dapagliflozin treatment at 5 mg/kg. Dapagliflozin treatment also markedly attenuated medial SMC loss. Though the difference was not significant, dapagliflozin treatment tended to attenuate CD8^+^ T cells and elastin degradation. Dapagliflozin treatment at 5 mg/kg caused a 53% reduction in neovessel density. Furthermore, dapagliflozin treatment mitigated further progress of existing AAAs.

**Conclusion:**

Dapagliflozin treatment ameliorated PPE-induced AAAs by inhibiting aortic leukocytes infiltration and angiogenesis.

## 1. Introduction

Abdominal aortic aneurysm (AAA) is a life-threaten vascular disease with approximately 80% mortality when ruptured [1, 2]. Although mechanisms by which AAAs form and progress remain incompletely understood, inflammation has been considered as a central player in AAA pathogenesis [3, 4]. Leukocytes, including T cells, macrophages, B cells, and mast cells play crucial roles in AAA disease [5–7]. Developing the strategies for lowering the incidence, or slowing the progression, of AAAs, is critical for effective disease management. Thus, pharmacological therapies for AAAs have attracted more attention in AAA research.

Diabetes is negatively associated with the formation and progression of clinical and experimental AAAs, with potential involvement of diabetes per se or diabetic medications [8–11]. Metformin, a widely used antidiabetic drug, suppressed experimental AAAs and reduced enlargement rate of clinical AAAs [11–15]. Other oral antidiabetic drugs, including sulfonylureas, thiazolidinediones, and dipeptidyl-peptidase 4 inhibitors, have also been reported to lower AAA incidence or attenuate AAA enlargement [16]. These previous studies suggest that some antidiabetes drugs may hold the potential to limit AAA progression.

Inhibitors to sodium-glucose transporter protein-2 (SGLT-2) for treating type 2 diabetes have been shown to reduce the risk for cardiovascular morbidities [17, 18]. SGLT-2 inhibitor reportedly suppressed aortic dissection and dilation [19]. However, questions remain whether SGLT-2 inhibition influences experimental AAAs. In a previous study, SGLT-2 inhibitor attenuated dissecting AAAs in hyperlipidemic mice following angiotensin II (Ang II) infusion [19]. Whether SGLT-2 inhibitors influence nondissecting AAAs in alternative AAA models such as elastase infusion model has not been investigated. The previous studies focused on the preventive effect of SGLT-2 inhibitors on AAAs by administering the inhibitor prior to AAA induction. Thus, it is not known whether the SGLT-2 inhibitor contains the progression of existing AAAs. In clinical trials, elective surgical AAA repair in patients with a diameter of 4.0-5.4 cm is not beneficial [20]. There is unmet clinical demand for innovating nonsurgical pharmacological therapies for limiting the continuous expansion and thus rupture of small AAAs [20, 21].

In this study, a SGLT-2 inhibitor dapagliflozin was administered to normoglycemic mice to evaluate the impact on AAA formation and progression induced by elastase infusion. Our study indicates that dapagliflozin treatment ameliorates the formation and progression of experimental AAAs partly by reducing aortic inflammation, neoangiogenesis, and preserving aortic structural integrity.

## 2. Materials and Methods

### 2.1. Mice

C57BL/6 mice were bred, maintained, and housed in the Laboratory Animal Center of Xi'an Jiaotong University, Xi'an, China. All experimental protocols were approved by the Laboratory Animal Administration Committee of Xi'an Jiaotong University (protocol No. 2019-1178). All animal experiments were conducted according to the Guidelines for the Care and Use of Laboratory Animals of Xi'an Jiaotong University.

### 2.2. Induction of Experimental AAAs

AAAs were created in male 10-12 weeks old C57BL/6 mice via intra-aortic infusion of porcine pancreatic elastase (PPE) freshly diluted in PBS for use (type I PPE, 1.5 units/mL, Table S1) as previously reported [22–26]. Briefly, mice were anesthetized by inhalation 2% isoflurane, abdominal laparotomy was created, and infrarenal aorta was exposed and infused with 30 *μ*L of PPE solution for 5 minutes under constant pressure. Thereafter, PE-10 tubing was withdrawn, aortomy was closed with 11-0 suture (Lingqiao, Ningbo, China), and aortic flow of lower limbs was restored. Following 2 hours postsurgical recovery, mice were housed in separate cages with free access to food and water.

### 2.3. Dapagliflozin Treatment

Dapagliflozin was purchased from Med Chem Express (Table S1) and freshly prepared by suspending in corn oil immediately prior to use. To test the effect on the formation of AAA, mice were daily treated with dapagliflozin at 1 or 5 mg/kg body weight through oral gavage (*n* = 10 mice for each dose). Additional 10 mice were treated with an equal volume (200 uL) of corn oil as vehicle. All treatments began 1 day before and terminated 14 days after PPE infusion. These doses have been proven effective in published studies [27–29]. To assess the effect on the progression of existing AAA, dapagliflozin (5 mg/kg) or an equal volume of vehicle was administered 4 days following PPE infusion, the time point by which aneurysms are formed in this model and continued for 10 days (*n* = 10 for each group) [30]. Fourteen days after PPE infusion, all mice were euthanized by carbon dioxide inhalation.

### 2.4. Aortic Diameter Measurement

After infrarenal aorta was exposed during surgery, abdominal aortae were immediately photographed under a microscope by a digital camera (ProS5 Lite, Motic, China) before PPE infusion. The baseline aortic diameters were measured with Images Plus 3.0 ML (Motic, China). Fourteen days following PPE infusion, aortic diameters were performed immediately prior to euthanasia. An aneurysm was defined as a more than 50% increase in aortic diameter over the baseline level [31].

### 2.5. Histochemical Analyses

On day 14 after PPE infusion, infrarenal aortae were harvested, embedded in O.C.T. media, sectioned (6 *μ*m) and acetone-fixed. Hematoxylin and eosin (H&E) and elastic van Gieson (EVG) staining were performed following standard or published procedure [32, 33]. Elastin degradation was graded using as previously reported [23–26, 32, 33]: grade I: elastin break or degradation was limited to one outer medial elastin layer; grade II: elastin degradation was involved in more than two layers, or entire medial elastin layer, but limited to less than 1/4 of the aortic circumference (AC); grade III: elastin degradation was involved in entire medial elastin layers, but limited to less than 1/2 the AC; and grade IV: elastin degradation was involved in entire elastin layers and expanded to more than 3/4 of AC [23–25].

### 2.6. Immunohistochemical Analyses

Tissue immunostaining was performed on acetone-fixed frozen sections using a standard 3-step immunoperoxidase procedure as described previously [25, 33]. Primary antibodies are antismooth muscle cells (SMCs) alpha-actin as well as rat monoclonal antibodies against CD68 (macrophages), CD4 (CD4^+^ T cells), CD8 (CD8^+^ T cells), B220 (B cells), and CD31 (blood vessel). T cells, B cells, and neovessels were quantified by counting positively stained cells or vessels per aortic cross-section (ACS). Medial SMC depletion and aortic macrophage accumulation were graded as I (mild) to IV (severe) as reported previously [31, 32]. Additionally, the expression of aortic matrix metalloproteins (MMPs: MMP2 and MMP9) was also evaluated by immunostaining. Secondary antibodies and other key reagents included biotinylated goat antirat antibody, donkey anti-goat IgG antibody, streptavidin-peroxidase conjugate, and AEC substrate kit. All primary and secondary antibodies used are summarized in Table S1.

### 2.7. Statistical Analysis

All data are expressed as the mean ± standard deviation (SD). The D'Agostino-Pearson omnibus normality test was used to determine whether individual datasets were normally distributed. For normally distributed data, one or two-way ANOVA analysis was conducted, followed by multiple comparisons for two group comparison. For data failing normal distribution, the nonparametric Kruskal-Wallis test was performed to compare three unmatched groups. For one time-point two group comparison, Student's *t* test or nonparametric Mann–Whitney tests were used to determine statistical difference for normally and nonnormally distributed data, respectively. All statistical analyses were performed by PRISM 7.0, and *p* < 0.05 was considered significant.

## 3. Results

### 3.1. Dapagliflozin Treatment Ameliorates Experimental AAAs

**Figure 1:**
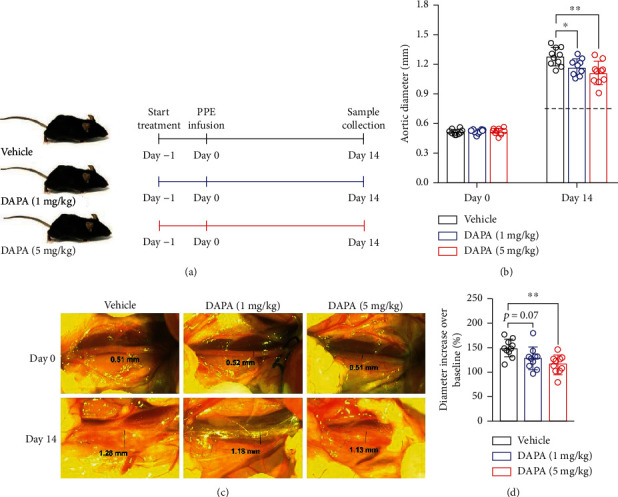
Dapagliflozin treatment suppresses experimental AAAs. (a) Study design: male C57BL/6 mice were given dapagliflozin (1 or 5 mg/kg body weigh) or vehicle by gavage for 14 days (*n* = 10 mice/group). (b) Mean and SD of aortic diameters at the baseline level (day 0) and 14 days after PPE infusion. Dotted line: the average aortic diameter following PBS infusion (0.8 mm). Two-way ANOVA followed by two group comparison, ^∗^*p* < 0.05 and ^∗∗^*p* < 0.01 between two groups. (c) Representative abdominal aortic images at the baseline (day 0) and 14 days after PPE infusion. (d) Change in aortic diameter (percent increase over the baseline level). One-way ANOVA followed by two group comparison, ^∗∗^*p* < 0.01 between two groups. DAPA: dapagliflozin.

To evaluate the ability of dapagliflozin to suppress experimental AAAs, mice were treated with 1 or 5 mg/kg body weight dapagliflozin or vehicle (Figure 1(a)). There was no difference in the baseline aortic diameter among three treatment groups (Figures 1(b) and 1(c)). Treatment with dapagliflozin at either dose significantly reduced aortic expansion following PPE infusion as compared to vehicle treatment especially at 5 mg/kg body weight (Figure 1). Dapagliflozin treatment at 1 and 5 mg/kg body weight led to 0.10 and 0.17 mm decreases in aortic diameters, respectively, as compared to vehicle treatment (Figure 1(c)). After subtracted an average aortic increase by PBS alone (0.8 mm) (Figure 1(b)), dapagliflozin treatment significantly reduced PPE-induced dilation (approximately 21% and 33% decreases in 1 or 5 mg/kg treatment groups, respectively). The change in aortic diameter was significantly smaller in mice treated with 5 mg/kg dapagliflozin than that in mice treated with vehicle (Figure 1(d)). However, the difference failed to reach statistical significance between 1 mg/kg dapagliflozin and vehicle treatment, although a reduced trend was noted (*p* = 0.07). These findings indicate that dapagliflozin treatment suppressed PPE-induced AAAs.

### 3.2. Dapagliflozin Treatment Preserves Medial SMCs

**Figure 2:**
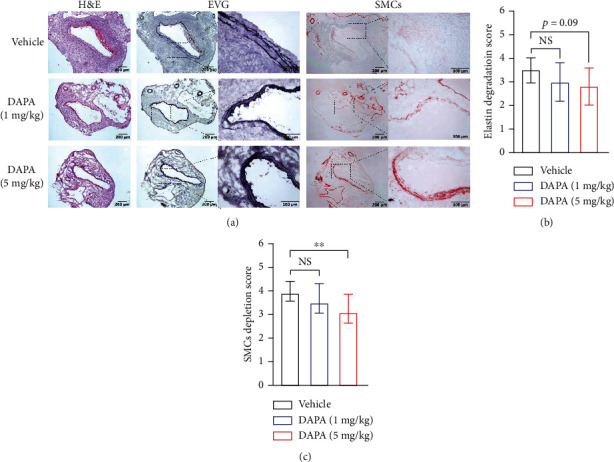
Influence of dapagliflozin treatment on medial elastin and SMC destruction. H&E, EVG, and SMC *α*-actin staining were performed on aortic frozen sections, graded, and semiquantified. (a) Representative aortic images for H&E, medial elastin EVG staining, and SMC immunostaining. (b) and (c) Mean and SD of elastin degradation (b) and SMC depletion (c) scores. Nonparametric Kruskal-Wallis test, ^∗∗^*p* < 0.01 between two groups, *n* = 10 mice in each group. DAPA: dapagliflozin; NS: not significant.

Medial elastin degradation and SMC loss are main histopathological features of AAAs. In Elastin and H&E staining, although we noted a trend for reduced medial elastin destruction in dapagliflozin-, as compared to vehicle-, treated PPE-infused mice, the difference was not statistically significant (Figures 2(a) and 2(b)). In SMC alpha-actin immunohistochemical staining, dapagliflozin treatment at 5 mg/kg body weight markedly attenuated medial SMC loss as compared to vehicle treatment (Figures 2(a) and 2(c)). These results indicate that antianeurysmal effects of dapagliflozin might be in part mediated by preserving medial SMCs.

### 3.3. Dapagliflozin Treatment Reduces Leukocyte Accumulation and the Expression of MMP2 and MMP9 in Aneurysmal Aorta

**Figure 3:**
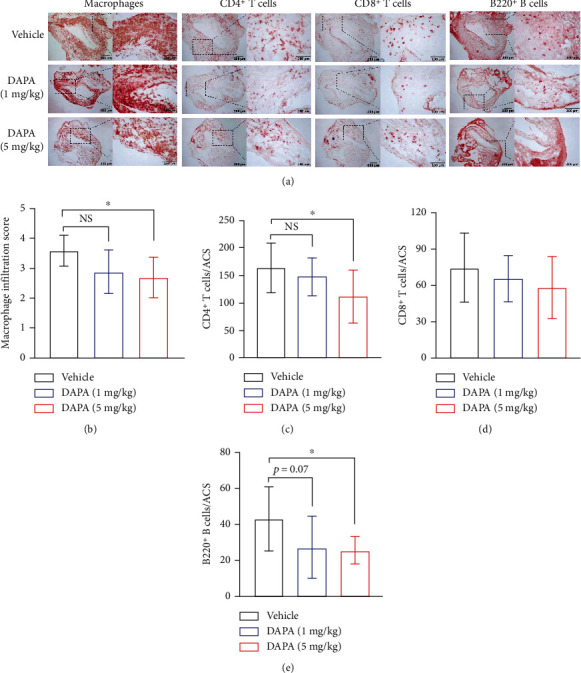
Dapagliflozin treatment attenuates aortic leukocyte infiltration. Immunostaining with antibodies against CD68, CD4, CD8, and B220 was used to evaluate the subsets of leukocytes in aneurysmal segment. (a) Immunostaining images of aortic macrophages, CD4^+^ T cells, and CD8^+^ T cells from PPE-infused mice treated with dapagliflozin or vehicle. (b)–(e) Quantification of different subsets of leukocytes per aortic cross-section (ACS) (mean and SD). For normally distributed data, one-way ANOVA analysis was conducted (T cells and B cells analysis), and for data failing normal distribution, the nonparametric Kruskal-Wallis test was performed (macrophage infiltration score). ^∗^*p* < 0.05 vs. vehicle. DAPA: dapagliflozin; ACS: aortic cross-section. NS: not significant.

**Figure 4:**
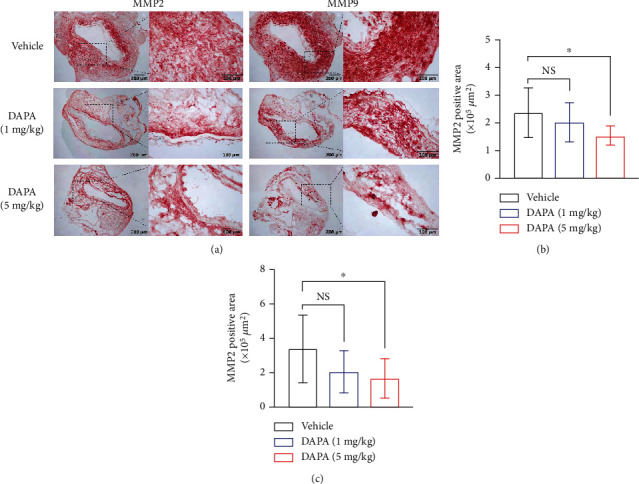
Dapagliflozin treatment reduced MMP2 and MMP9 expression levels. (a) Representative immunostaining images of MMP2 and MMP9 in aortae of PPE-infused mice treated with dapagliflozin or vehicle. (b) and (c) Quantification of MMP2 and 9 expression levels (mean and SD) in aneurysmal aorta. One-way ANOVA analysis was conducted. ^∗^*p* < 0.05 vs. vehicle. DAPA: dapagliflozin; NS: not significant.

Mural leukocyte accumulation is another histological hallmark and pathogenic determinant of AAAs [4–7]. Thus, we assessed the aortic infiltration of different subsets of leucocytes, including macrophages, T cells, and B cells by tissue immunostaining. In aneurysmal aorta, macrophages were the dominant infiltrating cells among all subsets evaluated (Figure 3). Compared with vehicle treatment, dapagliflozin treatment significantly reduced aortic macrophage infiltration especially at the high dose (Figures 3(a) and 3(b)). Similarly, dapagliflozin significantly reduced aortic CD4^+^ T cells and B cell accumulation (Figures 3(a), 3(c), and 3(e)). Although dapagliflozin treatment attenuated CD8^+^ T cells infiltration, there was no statistical difference between two treatment groups (Figures 3(a) and 3(d)). These results indicate that the suppression of experimental AAAs by dapagliflozin was in part mediated by inhibiting aortic macrophage accumulation. The expression of MMP2 and 9 was also diminished in aneurysmal aorta following dapagliflozin treatment as compared to vehicle treatment (Figure 4). MMPs, especially MMP2 and 9, were well known to involve in the development of AAA by regulated aortic wall remolding. The downregulation of both MMPs by dapagliflozin may be resulted from reduced MMP-producing inflammatory cell infiltration.

### 3.4. Dapagliflozin Treatment Suppresses Aneurysmal Angiogenesis

**Figure 5:**
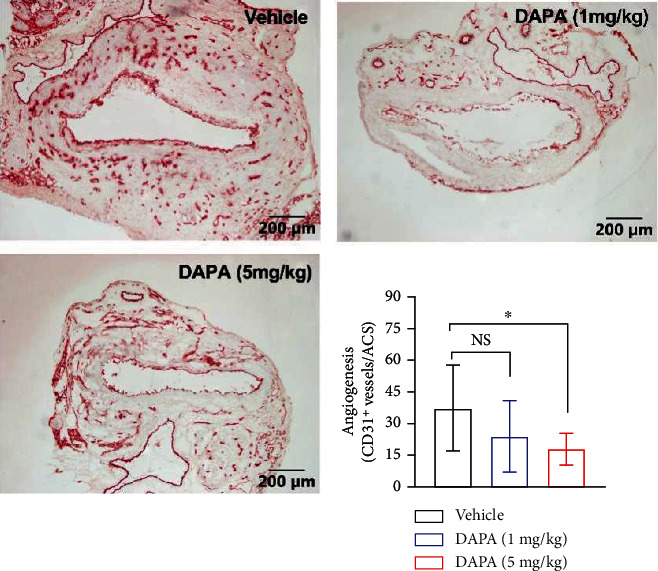
Dapagliflozin treatment attenuates aneurysmal angiogenesis. CD31 antibody staining was employed to identify neovessel in aneurysmal aorta. Quantification of mural neovessel as CD31-positive vessels per aortic cross-section. Data failing normal distribution, the nonparametric Kruskal-Wallis test was performed, ^∗^*p* < 0.05 between two groups, *n* = 10 mice in each group. ACS: aortic cross-section. DAPA: dapagliflozin; NS: not significant.

Because mural angiogenesis contributes to AAA pathogenesis, we determined whether the AAA suppression was associated with alteration in aortic wall angiogenesis by CD31 antibody immunostaining. Neovessel density was lower in dapagliflozin- than that in vehicle-treated aneurysmal aorta (Figure 5). In high-dose dapagliflozin-treated mice, there was a 53% reduction in neovessel density as compared to vehicle-treated mice (19 vs. 37 blood vessels/ACS). These results indicate that the suppression of mural angiogenesis may also contribute to AAA suppression by dapagliflozin.

### 3.5. Dapagliflozin Treatment Limits Further Progression of Existing AAAs

**Figure 6:**
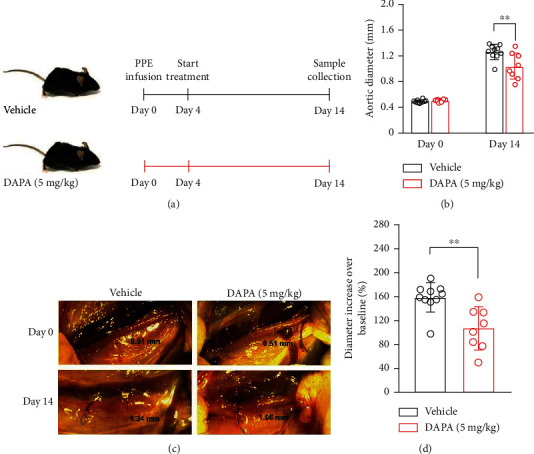
Dapagliflozin treatment limits further progression of existing AAAs. (a) treatment regimens. (b) Mean and SD of aortic diameters at the baseline level (day 0) and 14 days after PPE infusion. (c) Representative abdominal aortic images at the baseline (day 0) and 14 days after PPE infusion. (d) Change in aortic diameter (percent increase over the baseline level) following PPE infusions. Nonparametric Mann–Whitney test, ^∗∗^*p* < 0.01 compared with vehicle treatment, *n* = 10 (vehicle) or 8 (dapagliflozin at 5 mg/kg). DAPA: dapagliflozin.

**Figure 7:**
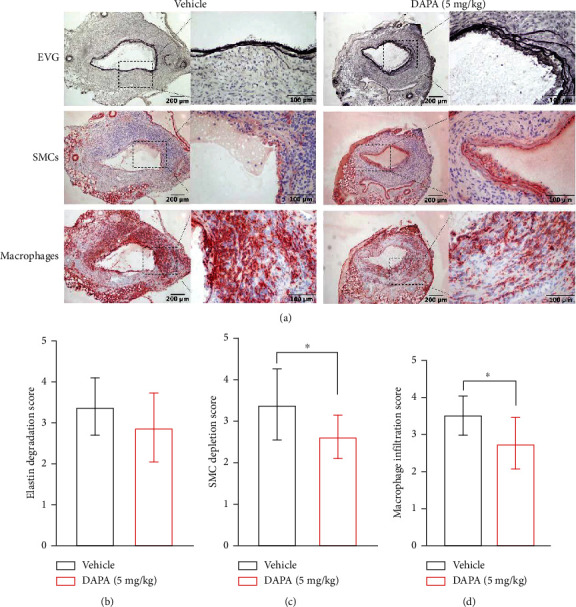
Influence of dapagliflozin on medial elastin, SMC destruction, and aortic macrophages infiltration on existing AAAs. EVG, SMC *α*-actin, and CD68 (macrophage) staining were performed on aortic frozen sections, graded, and semiquantified. (a) Representative aortic images for medial elastin EVG staining, SMC immunostaining, and aortic macrophages immunostaining. (b)–(d) Mean and SD of elastin degradation score (b), SMC depletion score (c), and macrophages in aneurysmal aortae. Nonparametric Mann–Whitney test, ^∗^*p* < 0.05 and ^∗∗^*p* < 0.01 compared with vehicle treatment, *n* = 10 (vehicle) and 8 (dapagliflozin at 5 mg/kg) mice. DAPA: dapagliflozin.

In order to verify its clinical value, dapagliflozin at 5 mg/kg was used to treat mice with existing AAAs (Figure 6(a)). In vehicle treatment, aortic diameters continuously increased to day 14. In contrast, dapagliflozin treatment reduced the progression of existing AAAs (Figures 6(b)–6(d)). In dapagliflozin treated mice, both aortic diameters and their data changes on day 14 were significantly smaller than that in vehicle-treated mice (Figures 6(b) and 6(d)). In histological analyses, dapagliflozin treatment reduced elastin degradation, preserved medial SMCs, and attenuated aortic inflammation (Figure 7). Though the difference was not significant, dapagliflozin treatment tended to prevent the further degradation of elastin in existing AAAs (Figures 7(a) and 7(b)). Dapagliflozin treatment also significantly inhibited macrophage infiltration and improved SMC cellularity as compared to vehicle treatment (Figures 7(a), 7(c), and 7(d)). Altogether, dapagliflozin treatment hindered further progression of aneurysmal expansion and histopathologies in mice with existing AAAs.

## 4. Discussion

Accumulating epidemiological studies have shown that SGLT2 inhibitors, a class of antidiabetic drugs, reduce the risk of atherosclerotic cardiovascular diseases possibly through glucose-independent mechanisms [17, 34–36]. Dapagliflozin is an FDA-approved SGLT-2 inhibitor for treating diabetes with demonstrated cardiovascular benefits in patients with diabetic renal dysfunction [37]. In patients with heart failure, dapagliflozin treatment slowed the progression of heart failure and reduced the risk of death from cardiovascular causes, regardless of diabetic status [36]. Abdominal aortic aneurysm seriously threatens life and health, and there is still a lack of effective drug therapy [21]. The negative association of antidiabetic drugs with AAA prevalence or growth has been reported [11, 16]. Although clinical data is not available, experimental studies have found that SGLT2 inhibitors suppressed angiotensin 2 induced aortic dissecting aneurysms [19]. Current widely used experimental AAA models include induction of nondissecting AAAs or a dissecting model contained intramural rupture [38]. However, the influence of SGLT2 inhibitors on nondissecting AAAs remains to be clarified. This study found that a SGLT-2 inhibitor, dapagliflozin, limited the progression of nondissecting AAAs in normoglycemic mice.

In this study, we first explored the effect of dapagliflozin on the initiation and progression of nondissecting AAAs by administration of the drug 1 day before PPE infusion. Dapagliflozin at either dose significantly reduced aortic dilation as compared to vehicle treatment especially at 5 mg/kg body weight. This finding is consistent with previous study, in which empagliflozin, an alternative SGLT-2 inhibitor, suppressed angiotensin II-induced dissection AAAs in ApoE deficient mice [19]. Elastin and SMCs are the main functional components of the vascular media. PPE infusion severely degrades elastin and causes SMC apoptosis leading to media SMCs depletion [25, 39]. In PPE infusion model, aortic leukocytes produce inflammatory cytokines, which may promote SMC apoptosis or dedifferentiation and show less alpha-actin positive staining. Dapagliflozin treatments significantly attenuated medial SMC loss and showed a trend for the preservation of elastin as compared to vehicle treatment. Another SGLT-2 inhibitor canagliflozin has been shown to inhibit intracellular glucose metabolism and promote autophagy critical for cell survival under various stress conditions [40]. We thus postulate that AAA suppression by dapagliflozin SGLT-2 inhibitor may be potentially mediated by modulating autophagy or apoptosis [41, 42]. Another explanation of protective effect of dapagliflozin treatments on SMCs and elastin may be associated with the inhibition of aortic inflammation. Dapagliflozin treatment significantly reduced aortic leucocytes infiltration, including macrophages, T cells, and B cells, following PPE infusion in mice. These results suggest that the suppression of experimental AAAs by dapagliflozin was also in part mediated by inhibiting aortic wall macrophage accumulation. SGLT-2 inhibitors have been shown to reduce the expression of pro-inflammatory cytokines, NLRP3, MyD88, and NF-*κ*B [19, 43, 44], a transcription factor regulating multiple inflammatory process. SGLT-2 inhibitors promoted anti-inflammatory macrophage activation by modulating reactive oxygen and nitrogen species-dependent STAT3 pathway [45]. After intraluminal PPE infusion, the inflammation caused by it dominates the progression of AAA. Infiltrated aortic leukocytes produce elastin-degrading enzymes which may be responsible for the medial elastin degradation [46]. The suppressive effect of aortic leukocyte infiltration by dapagliflozin may also contribute to protect the elastin degradation. Macrophages contribute to the pathogenesis of AAA by producing MMPs. MMPs including MMP 1, 2, 3, 9, 12, and 13 were elevated in the aortic wall of AAA [47]. Dapagliflozin at 5 mg/kg reduced the expression levels of aortic MMP2 and MMP9, which might promote SMC survival in response to PPE infusion. AAAs are also associated with an abnormal angiogenesis as demonstrated by anti-VEGF-A therapy in experimental AAAs [24, 48]. In this study, it was found that dapagliflozin treatment was able to reduce arterial adventitia angiogenesis. The interaction of abnormal angiogenesis and other inflammatory responses may be attenuated by dapagliflozin. Taken together, suppressing aortic inflammation including mural anagiogenesis may be major mechanisms by which dapagliflozin suppresses experimental AAAs in the PPE-induced AAA model.

To further evaluate the translational value of dapagliflozin in AAAs, the effect on existing AAA was investigated. Usually, aneurysms are formed after 4 days following PPE infusion in mice [30]. Similar with pretreatment experiment, posttreatment of dapagliflozin significantly limited the expansion of abdominal aortic diameter in PPE-infused mice. These findings indicate that dapagliflozin confined the progression of existing AAAs and may hold the potential for clinical AAA management. The inhibition of aortic inflammation and SMC-protective effect may play important role in this process. All the above results suggest that administration of dapagliflozin in diabetic patients, like the use of metformin, may contribute to the negative association between diabetes mellitus and AAA prevalence and growth [11, 15]. Therefore, to investigate the protective effect of dapagliflozin on aneurysms, further clinical l studies are needed.

Our study has some limitations. First, this study was carried out in normoglycemic mice, and dapagliflozin treatment has no recognizable effect on random nonfasting blood glucose level (Figure S1). Because AAA was substantially suppressed in diabetic mice [26, 49], it is impractical to prove whether dapagliflozin causes further protection against AAAs in hyperglycemic conditions. Second, neutrophils are critical at the early stage of experimental AAAs [50]. Because we collected aortic specimens on day 14 following PPE infusion, neutrophils were not included in this study.

In conclusion, this study found that dapagliflozin treatment ameliorated elastase-induced experimental nondissecting AAAs in part by attenuating mural macrophage accumulation and neoangiogenesis. In addition to its benefits for atherosclerotic cardiovascular diseases, our research suggests that treatment with SGLT-2 inhibitors such as dapagliflozin may reduce the risk, and the progression of small AAAs.

## Data Availability

Data is available on request.

## Supplementary Materials

Supplementary Materials Table S1: reagents. Figure S1: the effect of dapagliflozin treatment on random blood glucose in nondiabetic mice.
