# Supplementary material for: Dapagliflozin Ameliorates the Formation and Progression of Experimental Abdominal Aortic Aneurysms by Reducing Aortic Inflammation in Mice
Source: Oxid Med Cell Longev. 2022 Jan 28;2022:8502059. doi: 10.1155/2022/8502059 (PMC8816542; doi:10.1155/2022/8502059)
Supplement: Supplementary Materials — Table S1: reagents. Figure S1: the effect of dapagliflozin treatment on random blood glucose in nondiabetic mice. [file 8502059.f1.docx]

Table S1. Reagents

| Antibodies | | | | |
| --- | --- | --- | --- | --- |
| Target antigen | Catalog # | Dilution ratio | Host | Source |
| Mouse SMC alpha actin | NB300-978 | 1:200 | Goat | Novus Biologicals |
| CD68 | 137002 | 1:200 | rat | Biolegend |
| CD4 | 100402 | 1:200 | rat | Biolegend |
| CD8 | 100702 | 1:200 | rat | Biolegend |
| B220 | 103202 | 1:200 | rat | Biolegend |
| CD31 | 100402 | 1:200 | rat | Biolegend |
| MMP2 | AF1488 | 1:200 | Goat | R&D Systems |
| MMP9 | AF909 | 1:200 | Goat | R&D Systems |
| anti-rat antibody | BA-9400 | 1:400 | goat | Vector Laboratories |
| anti-goat IgG | 705-065-003 | 1:400 | donkey | Jackson Immuno Research |
| Additional reagents | | | | |
| Name | Catalog # | Dilution ratio | Host | Source |
| Elastase | E-1250 | 1:20 | Porcine pancreas | Sigma-Aldrich Corp |
| Dapagliflozin | HY-10450 | NA | NA | Med Chem Express |
| streptavidin-peroxidase conjugate | 016-030-084 | 1:400 | NA | Jackson Immuno Research |
| AEC substrate kit | SK-4200 | NA | NA | Vector Laboratories |

NA, not applicable.


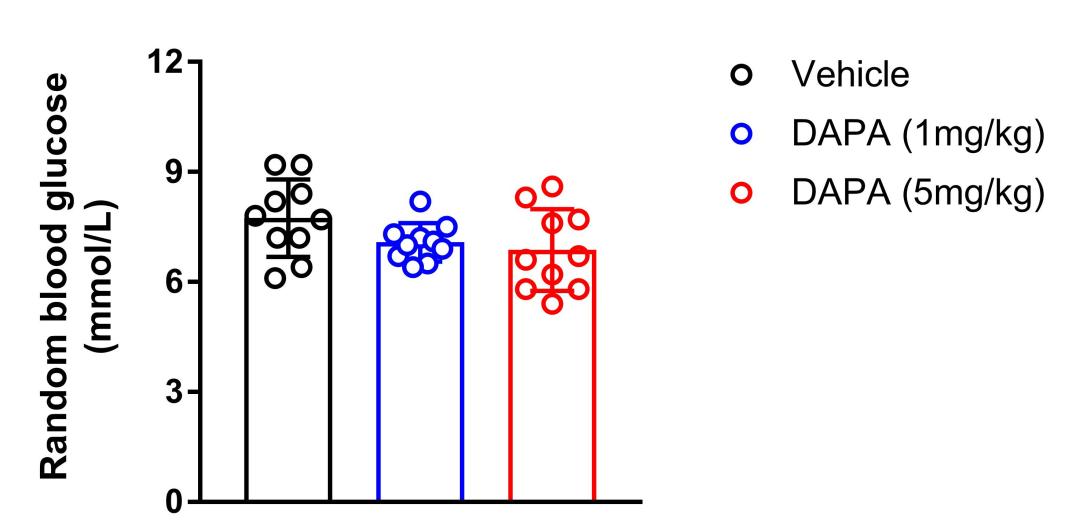


Figure S1. The effect of dapagliflozin treatment on random blood glucose in non-diabetic mice. The data are expressed as the mean±SD, n=10 mice in each group.
